# The Maternal Diet Index and Offspring Microbiota at 1 Month of Life: Insights from the Mediterranean Birth Cohort MAMI

**DOI:** 10.3390/nu16020314

**Published:** 2024-01-20

**Authors:** Raúl Cabrera-Rubio, Kaci Pickett-Nairne, Sonia González-Solares, Maria Carmen Collado, Carina Venter

**Affiliations:** 1 Institute of Agrochemistry and Food Technology, National Research Council (IATA-CSIC), Agustin Escardino 7, 46980 Paterna, Spain; mcolam@iata.csic.es; 2 Department of Pediatrics, University of Colorado School of Medicine, University of Colorado, Aurora, CO 80045, USA; kaci.pickett-nairne@cuanschutz.edu (K.P.-N.); carina.venter@childrenscolorado.org (C.V.); 3 Department of Functional Biology, University of Oviedo, 33006 Oviedo, Spain; soniagsolares@uniovi.es; 4 Diet, Microbiota and Health Group, Instituto de Investigación Sanitaria del Principado de Asturias (ISPA), 33011 Oviedo, Spain

**Keywords:** maternal diet in pregnancy, infant microbiome, nutrition

## Abstract

**Background:** Maternal diet during pregnancy may play a role in infant health outcomes via the maternal microbiota. We assessed the association of the maternal diet index for the Mediterranean area (MDI-med) with infant gut microbiota at 1 month of life. **Methods:** The MAMI study is a longitudinal birth cohort in the Mediterranean area. In this work, a cross-sectional study, including 120 mother–infant dyads with available maternal diet and infant microbiota at 1-month-old data, was undertaken. The MDI developed in the US (MDI-US) was adapted for the MAMI cohort (MDI-med). Stratification based on extreme values resulted (22 in the “lower” MDI-med group and 23 in the “upper” group from the mean). Relative microbial abundances and alpha (microbial richness and diversity indexes) and beta diversity (Bray–Curtis distance matrix) were compared between the groups. **Results:** Higher maternal daily vegetable intake and lower red meat intake were the characteristics of the “upper” MDI-med group. Significantly lower microbial diversity (Shannon and InvSimpson index (*p =* 0.01)), but no changes in richness (Chao1 index) nor in beta-diversity, using Bray–Curtis distance, were observed in the “upper” group, compared to the “lower” MDI-med group. A higher relative abundance of the *Bifidobacterium* genus (Actinomycetota phylum) was associated with maternal daily vegetable and yogurt intake. **Conclusion:** Reduced infant microbial diversity at 1 month of age was associated with “upper” MDI-med scores. Higher maternal intakes of vegetables and yogurt were associated with higher relative abundances of the *Bifidobacterium* genus in the infant gut. Further studies are needed to understand the link between pregnancy diet, infant microbiota, and health outcomes.

## 1. Introduction

The maternal diet during pregnancy plays a role in health outcomes in the infant [1]. Maternal diet may affect the infant microbiome either directly [2] or indirectly, via the maternal microbiome [3]. It is known that the infant gut microbiome is associated with a range of offspring health outcomes [4]. A small number of studies show the effect of nutritional intake during pregnancy on the infant gut microbiota [2,5].

We have recently shown that a novel maternal diet index (MDI-US) during pregnancy was associated with reduced outcomes of asthma, wheezing, allergic rhinitis, and eczema in the child, up to 4 years of age [1]. The MDI-US included weighted measures of seven dietary components: vegetables, yogurt, fried potatoes, rice/grains, red meats, pure fruit juice, and cold cereals. The results in the US population showed that vegetables and yogurt were identified as protective factors for total allergy risk, while the other components were recognized as risk factors. A study from Taiwan [6] showed that infant gut microbiomes at 2 months of age clustered differently for high and low maternal fruit and vegetable consumption (*p* < 0.001), with species-specific differences also noted between the two groups. 

Low-fiber cereals/grains, fatty fried foods, and high sugar foods were given a negative score in the MDI-US. These dietary components, closely linked to Westernized lifestyles, have been associated with reduced gut microbiome diversity and function [7]. During pregnancy, a lower maternal intake of fiber-rich foods has been associated with reduced numbers of short chain fatty acid (SCFA)-producing bacteria [8]. Fat intake in pregnancy was also positively correlated with Firmicutes, and protein, but the fiber intake were negatively correlated with Firmicutes at birth [3].

The aim of this study was to determine maternal diet intake during pregnancy, adapting the MDI-US into the Mediterranean Maternal Diet Index (MDI-med), to study the association between the MDI-med and infant gut microbiota diversity and composition at 1 month of life. The information derived from this work could be useful in deepening our knowledge between maternal dietary intake in pregnancy and infant microbiomes.

## 2. Materials and Methods

### 2.1. Study Participants and Clinical and Demographic Characteristics

A subsample of the MAMI cohort included 120 mother–infant dyads with a maternal diet information and infant microbiota dataset at one month. The MAMI cohort is a prospective mother–infant cohort in the Spanish–Mediterranean area [2,9]. Nutritional, anthropometrical, and clinical parameters of mother–infant pairs were recorded. Available data included maternal age and education status, pre-gestational body mass index (BMI), and weight gain during pregnancy. Infant sex, gestational age, the mode of delivery (C-section vs. vaginal), intrapartum antibiotic exposure, and neonatal weight and length at birth were also collected. Some participants were exposed to antibiotics during pregnancy and this information was also available. Infant feeding at 1 month of age was recorded and categorized as breastmilk only, mixed feeding, or formula only. 

### 2.2. Dietary Information

Dietary information was obtained using a semi-quantitative food consumption questionnaire (FFQ), which was completed during a personal interview. Items were grouped into food groups according to the Centro de Enseñanza Superior de Nutrición Humana y Dietética (CESNID) categorizations [2]. Women were asked to fill out this 140-item questionnaire about their diet during pregnancy within the first week after delivery [10]. Answers to the questionnaire relating to intake of vegetables, yogurt (containing live cultures and no sugar in the MAMI study), French fries, rice/grains and low-fiber grains, red meats, 100% pure fruit juice (raw fruit was not included in the index) and cold cereal were subsequently selected, to create a maternal diet index for the MAMI participants (MDI-med), similar to the maternal diet index (MDI-US) of the Healthy Start cohort. The foods and food groups included for analysis of the MDI-US and the foods used for the Spanish FFQ to create the adapted MDI-med are listed in Appendix A. 

The MDI-med was built based on the frequency a person reported they consumed each of these categories, i.e., data were given for how many times per day, week, month, and year each food item was used. Any frequency was converted to servings per day and then put into a weighted equation built for the Healthy Start cohort [1]. Once the MDI-med was computed for all dyads, two groups based on the extremes of MDI-med values (“upper” and “lower” MDI-med value groups) were assigned for this analysis, as previously implemented [11]. Extremes were obtained using the mean ± one standard deviation (SD) from the cohort mean; thus, samples above the mean +1SD were included in the “upper” group, while samples below the mean −1SD were included in the “lower” group.

### 2.3. Infant Microbiota at 1 Month

Infant fecal samples 1 month after birth were collected in sterile containers by the parents at home using detailed instructions. Samples were immediately stored at −20 °C, transported within 24 h of collection, and stored at −80 °C until analysis [3]. In brief, infant fecal samples were processed and the obtained DNA was used for amplification of the V3–V4 variable region of the 16S rRNA gene, which was sequenced using the Illumina platform, as detailed previously [12]. Quality filtering, sequence joining, and chimera removal were achieved using the DADA2 pipeline. Taxonomic assignment was performed using the Silva v138 database, with an accuracy of 80%. Samples with low relative abundance (<0.01%) and those present less than five times in at least 20% of the samples were filtered. Samples with less than 1000 reads (*n* = 5) were also removed from the final data analysis.

### 2.4. Statistical Analysis

RStudio (R version 3.6.3 or 4.1.2) was used for all analyses [2]. Hypothesis testing was conducted at an alpha level of 0.05. Where necessary, the *p*-values were adjusted by the false discovery rate (FDR) method, applying Benjamini and Hochberg. *p* < 0.05 was considered statistically significant, although *p*-values < 0.08 were also reported as a trend.

#### 2.4.1. Cohort Description and Clinical Data

Descriptive statistics were calculated for maternal and infant characteristics, including means (standard deviations) or medians (interquartile range; IQR) for continuous variables, depending on distribution and frequencies, and percentages for categorical variables. Continuous variable differences were assessed using *T*-test and Mann–Whitney analyses, according to data normality, assessed by the Shapiro–Wilk test. A chi-squared test was used to assess the significance of the differences in the population’s categorical variables. To assess the association between individual MDI-med components and the two MDI-med groups (“upper” and “lower” groups, separated by the median of the MDI-med index), an variance analysis test (ANOVA test) was used to compare the daily intake of each component between the groups.

#### 2.4.2. Alpha Diversity Analysis

Alpha diversity metrics summarize the structure of a microbial community, with respect to its microbial richness and diversity. The calculation of the microbial alpha diversity included the data on richness (Chao1) and diversity (Shannon and InvSimpson) indices for each sample, obtained by the *phyloseq* R package [13]. The differences by group were assessed by ANOVA tests. To determine the association between the MDI-med index and the alpha diversity of 1-month-old infant microbiota, separate general linear regression models were fit for the alpha diversity indices, Chao1, the Shannon index, and the InvSimpson index, with the binary diet group as a predictor. An unadjusted model was performed, with the maternal diet index as the only predictor, as well as adjusted models with a priori selected covariates. The initial adjusted model (model 1) included the mode of birth and antibiotic exposure. An additional adjusted model was built (model 2), which included breastfeeding type at 1 month of age (breastmilk, formula, or mixed) and maternal gestational antibiotic use, as these have been associated with microbial diversity in some studies [14,15,16]. The final models report, where appropriate, beta estimates, *p*-values, and 95% confidence intervals for the associations.

#### 2.4.3. Beta Diversity Analysis

Amplicon sequence variants (ASVs) were used to carry out the beta diversity analysis. The permutational multivariate analysis of variance test (PERMANOVA test) (adonis2) from the vegan R package [17] was used to evaluate the overall differences in microbiota structure between MDI-med groups, using multivariate dispersions (nonmetric dimensional scaling (NMDS)). In addition, we also performed a principal component analysis (PCoA) with the Bray–Curtis matrix. The data were then normalized by log10 transformation (to avoid infinite values of the logarithm) and significant differences were adjusted by FDR method, applying Benjamini and Hochberg. *p* < 0.05 was considered statistically significant, although *p*-values < 0.08 were also reported as a trend.

#### 2.4.4. Individual (MDI-med) Components and Infant Microbiota

A redundancy discriminant analysis (RDA, *microeco* R package) was also performed, to assess the relationship between microbiota at the genus level and the MDI-med components. It was also used to visualize these differences according to the MDI-med group, while projecting the main diet variables and dominant genus vectors. Spearman correlations were used to assess the linear relationship between the top 40 main taxa and the individual MDI-med components.

Random forests were used to determine the most important beta diversity factors for predicting the differences between the “upper” and “lower” dyads of the MDI-med (*RandomForest* R package, version 4.6-14). To train the multivariable statistical models for the prediction of the dyads’ MDI-med group, we first removed taxa with low abundance and overall prevalence (abundance cutoff: 0.001). The features were then normalized by log10 transformation (to avoid infinite values of the logarithm, a pseudo count of 1 × 10^−5^ was added to all values), followed by standardization as centered logarithmic ratio (log.clr). Data were randomly partitioned into test and training sets in a repeated tenfold cross-validation. The trained model was then used to predict the skipped test set, and, finally, all predictions were used to calculate the area under the operating characteristic curve for each variable (AUROC).

## 3. Results

### 3.1. The Maternal Diet Index (MDI)

For all dyads with maternal diet and infant microbiota information (N = 120), the median MDI-med score was 73.74 (IQR 72.4, 75.6), with a minimum of 67.2 and maximum of 81.95. This was higher (*p* > 0.05), and the values were wider spread, than the MDI-US scores in Healthy Start cohort (N = 1253), which had a median MDI-med of 72.05 (IQR 71, 73) with a minimum of 63.7 and a maximum of 76.5. Our “lower” diet index group (N = 23) was defined as having an MDI-med value that was below the average MDI-med value for the individuals below the median of all dyads (median of N = 120: 73.74; average of N = 60 below median: 71.94). The “upper” diet index group (N = 22) was defined based on reaching a threshold calculated based on a theoretical “perfect” diet based on US Dietary guidelines [18] and were required to have an MDI-med value above 76.35 (Appendix B). Our resulting analytic cohort size was, thus, *n* = 45 (Table 1) and thedemographic information on the total sample size of *n* = 120 is shown in Appendix C. No statistical differences were found between those included and those excluded.

### 3.2. Clinical Characteristics and Descriptives

The overall sample of 45 dyads included pairs with maternal diet data in the two MDI-med groupings and 1-month-old infant microbiota. No significant differences were found between maternal and infant anthropometrical and clinical parameters based on MDI-med group during pregnancy (Table 1), other than neonatal length at birth. Dyads where the mother had a “healthier” MDI-med (“upper” group) showed no significant differences from those with an “unhealthier” MDI-med (“lower” group), except for a slightly larger length at birth in the “upper” group (mean 51 cm vs. 49 cm; *p* = 0.002). The overall distribution of the MDI-med values in the “lower” group had a mean of 70.59, with a minimum index value of 67.19 and a maximum of 71.93, while the “upper” group had a mean of 77.34 (min 76.35, max = 81.95).

**Figure 1:**
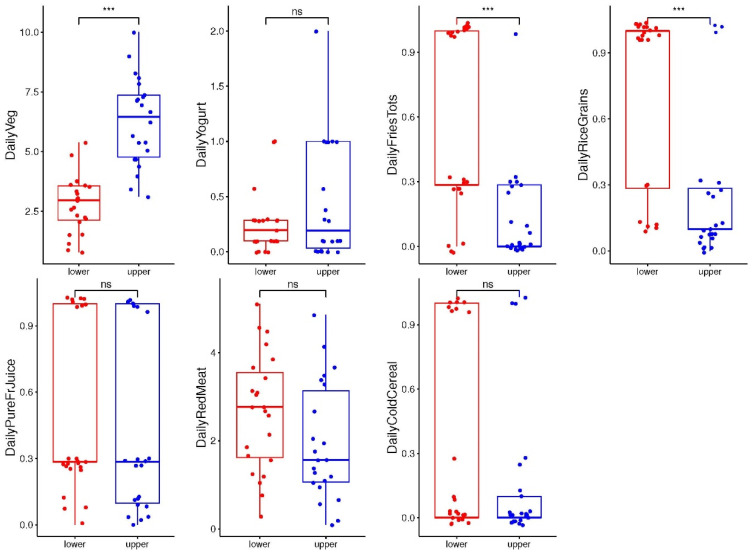
ANOVA analysis of the serving intake of components of the MDI (Y axis) vs. the MDI-med group (“lower” and “upper” groups; X axis) (adjusted FDR analysis). Figure legend: FDR: false discovery. “ns”: no significant. *** *p*-value < 0.001).

We analyzed the importance of the association between the individual MDI-med components and the different groups of the MDI-med, with daily vegetable intake (*p* = 0.001) being significantly higher in the “upper” group, and fried potato products (*p* = 0.001) and carbohydrate intakes (mainly rice and low-fiber grains, *p* = 0.001) being significantly higher in the “lower” group (Figure 1). No significant differences were reported for the other diet components studied.

**Figure 2:**
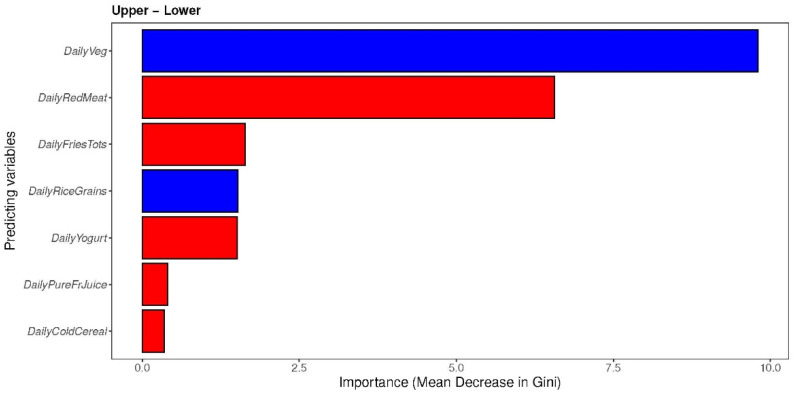
Random forest (RF) analysis indicating which specific dietary factors in the MDI-med were associated with each diet group, i.e., the “upper” and “lower” MDI-med groups (adjusted FDR analysis). Figure legend: FDR: false discovery rate; red variables were highly associated with the “upper” group (healthier), whereas blue variables were highly associated with the “lower” group (less healthy).

Finally, the prediction of factors that contribute to the different groups of the MDI was performed, using a random forest classifier. The random forest plots (Figure 2) show that the important predictors associated with the “upper” group are those related to daily vegetable intake (*DailyVeg*) and, for the “lower” group, they are the ones related to daily red meat intake (*DailyRedMeat*) and daily fries intake; all of these are described from greatest to least importance using mean decrease in Gini.

### 3.3. Infant Microbiota at 1 Month

A total of 2,285,937 good quality sequences were obtained from the subjects included in the study, after bioinformatics processing (including length/quality filtering and chimera removal) and the removal of contaminant sequences, according to the criteria described in Materials and Methods. This represents a median number of 50,798.6 sequences per sample (±9199.812). An average of 5079.6 sequences, and 160.61 ASVs were extracted from these sequences.

#### 3.3.1. Association between the Maternal Diet Index and Infant Alpha Diversity Indices (Shannon Index, Chao1 Index, and InvSimpson Index)

**Figure 3:**
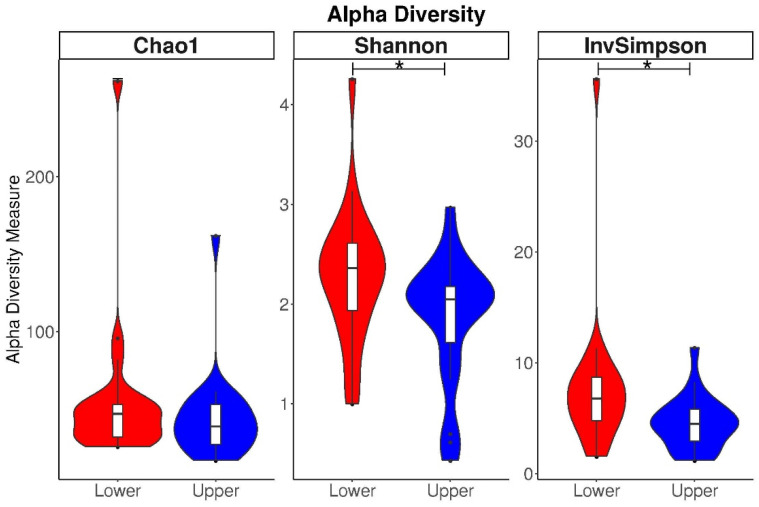
Boxplots of distributions of alpha diversity measures by MDI-med groups (“upper” and “lower” MDI-med scores) in the adjusted models. Figure legend: * *p*-value < 0.05 indicates significant differences between groups, based on one-way ANOVA.

The alpha diversity showed significant differences between the “upper” and “lower” groups, when using a one-way ANOVA (Figure 3), calculated as the Shannon and InvSimpson indices in infants (*p =* 0.01). The Chao1 index showed a similar trend but did not show any significant differences. Thus, in general, the infant microbiota from the “upper” MDI-med group were characterized by lower microbial diversity and richness than the “lower” MDI-med group.

Additionally, using linear regression models (unadjusted and adjusted), the two groups, based on MDI-med index in pregnancy, showed significantly different alpha diversity scores in the infant microbiome at 1 month when using the Shannon and InvSimpson indices, but not the Chao1 index, in the unadjusted models (Table 2).

After adjusting for the mode of birth, the Shannon index of the one-month-old infant microbiota was estimated to be reduced by 0.33 times (beta −0.33) in the “upper” MDI-med group, compared to the “lower” MDI-med group (95% CI −0.66, −0.01; *p* = 0.04), and the inverse Simpson index was also reduced (beta −1.78 ) for the “upper” group (95% CI −3.62, 0.07; *p* = 0.06). After additionally adjusting for breastfeeding and gestational antibiotics use, there were no longer any significant differences between the groups (*p* > 0.05), although some trends in the Shannon index in model 2 (*p* = 0.07) and the inverse Simpson index in model 1 (*p* = 0.06) were observed. Thus, alpha diversity has an impact on the Shannon and inverse Simpson indices of the infant microbiota between the “lower” and “upper” groups of the MDI-med factor, after adjusting for the mode of delivery and for breastfeeding in the first model and gestational antibiotics use in the second.

#### 3.3.2. Association between the Maternal Diet Index and Beta Diversity (Bray–Curtis) of Infant Microbiomes at 1 Month

**Figure 4:**
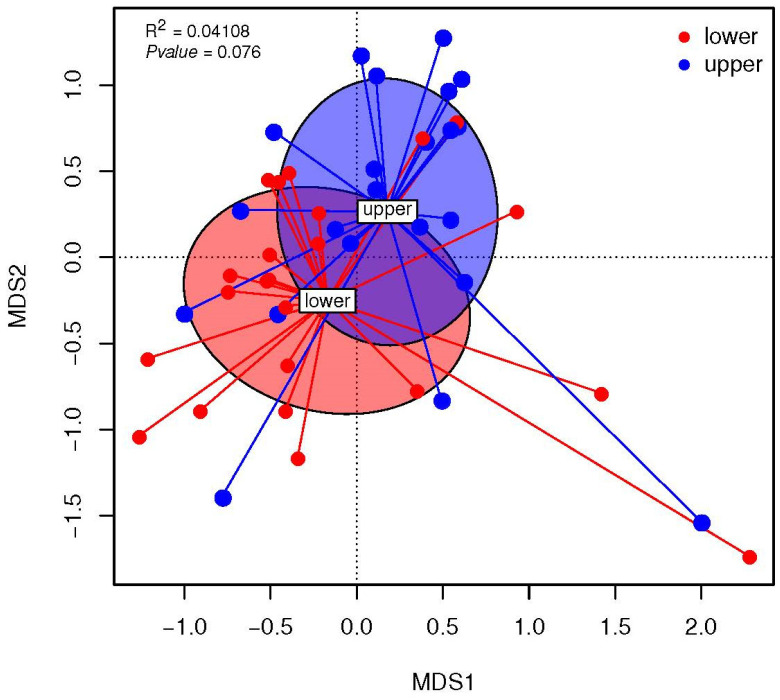
Multivariate dispersions (nonmetric dimensional scaling (NMDS)) of the Bray–Curtis dissimilarity distances of 1-month-old infant microbiota by dichotomized MDI-med group (FDR adjusted analysis).

Two distinct, trend but not significant, infant gut microbial communities were observed between the “upper” and “lower” MDI-med groups (Figure 4, PERMANOVA R^2^ = 0.041, *p* = 0.076). The other strongest factors were the mode of delivery and lactation, for beta diversity described with the Bray–Curtis index. The mode of delivery explained the variability of the beta diversity (R^2^ = 0.036, *p* = 0.031 PERMANOVA) and lactation also explained the variability of the beta diversity (R^2^ = 0.055, *p* = 0.048 PERMANOVA). Therefore, the contributing factors to infant microbial variation at 1 month of life are the mode of delivery, lactation type, and maternal MDI-med, which explain the differences found in the beta diversity analysis.

**Figure 5:**
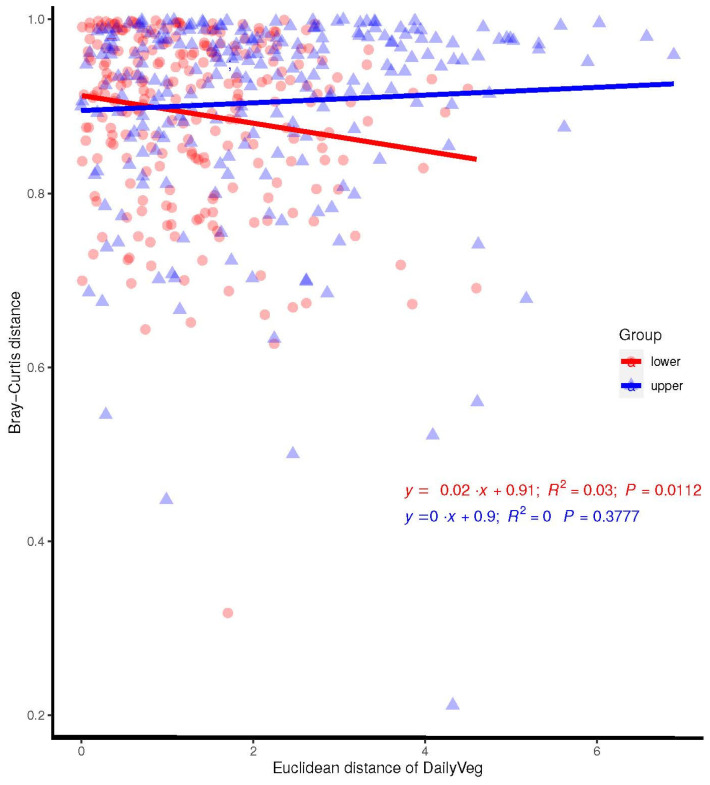
Scatterplot of Bray–Curtis distances and Euclidian distance of daily vegetable intake, with best fit lines and correlations for MDI groups (adjusted analysis).

Combining lactation and the MDI-med clusters in the joint PERMANOVA analysis showed a significant association (R^2^ = 0.032, *p* = 0.037). Comparing Euclidian distance and Bray–Curtis distance showed a significant association between vegetable intake in the “lower” group and beta-diversity, even after adjusting for the mode of birth, breastfeeding, and antibiotic use during pregnancy (Figure 5, R^2^ = 0.030; *p* = 0.011).

#### 3.3.3. The Associations between Maternal Diet Index and Individual Infant-Specific Microbiota

**Figure 6:**
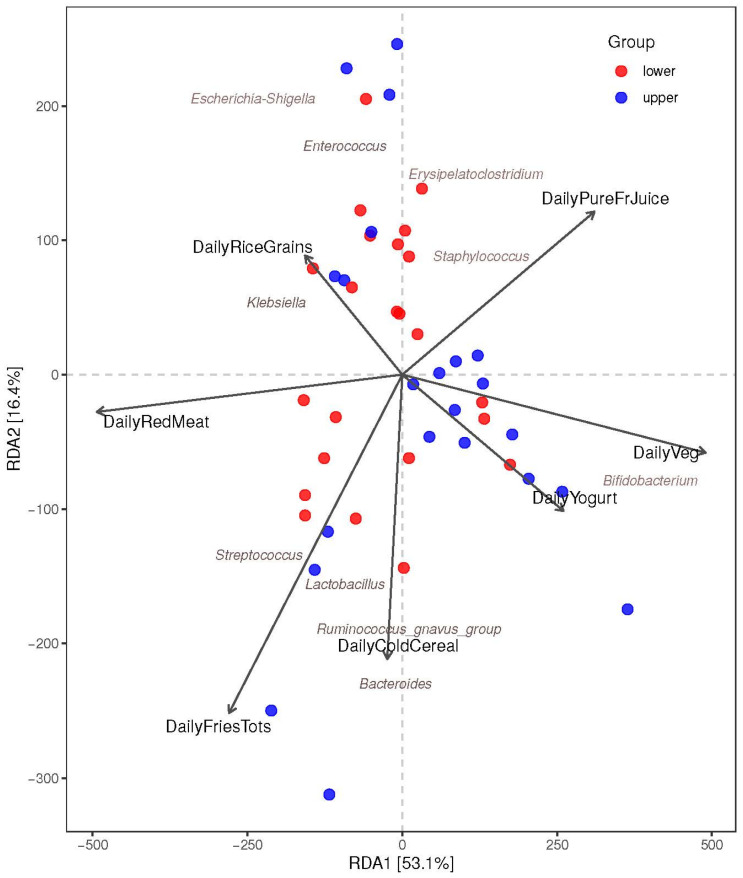
Biplot of the redundancy discriminant analysis (RDA) of infant 1-month genus abundance vs. the components of the MDI-med. Individuals in each MDI-med group are indicated by colored points (*p*-values are from adjusted FDR analysis). Figure legend: FDR: false discovery rate.

While no significant associations (*p* > 0.05) between the MDI-med “upper” and “lower” groups and specific microbes were found, there was a similar trend in the RDA between *Bifidobacterium* (Actinomycetota phylum) and daily vegetable intake (*p*-value = 0.032 and FDR *p*-value = 0.085), as well as daily yogurt intake (FDR *p* = 0.770). There was also an indication of a similar direction between carbohydrate intake (mainly daily rice and low-fiber grains) and *E.coli/ Shigella* (*Enterobacteriaceae* family) and *Enterococcus* (*Enterococcaceae* family). Daily red meat, fried potato products, and cold and low-fiber cereal were associated with *Streptococcus*, *Lactobacillus, Ruminococcus gnavus*, and *Bacteroides* groups. Daily pure fruit juice was associated with *Erysipelatoclostridium* and *Staphylococcus* genus (Figure 6).

**Figure 7:**
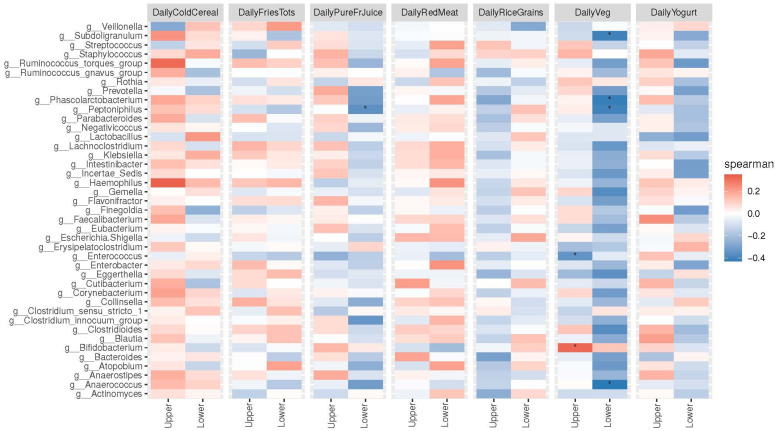
Spearman correlation heat map between 40 dominant taxa and MDI-med components stratified by MDI-med group (adjusted FDR analysis). Figure legend: FDR: false discovery rate. * *p*-value < 0.05 indicates significant differences between groups.

The Spearman rank correlations (Figure 7) showed significant positive correlations, particularly for daily vegetable intake in the “upper” group with the genera *Bifidobacterium* (*p* = 0.01), and significant negative correlations in the “upper” group with *Enterococcus* (*p* = 0.01). In the “lower” group”, daily vegetable intake showed significant negative correlations with the genera *Anaerococcus* (*p* = 0.01), *Peptoniphilus* (*p* = 0.01) *Phascolarctobacterium* (*p* = 0.01), and *Subdoligranulum* (*p* = 0.01). Daily pure fruit juice intake also showed a negative association with *Peptoniphilus* (*p* = 0.01).

**Figure 8:**
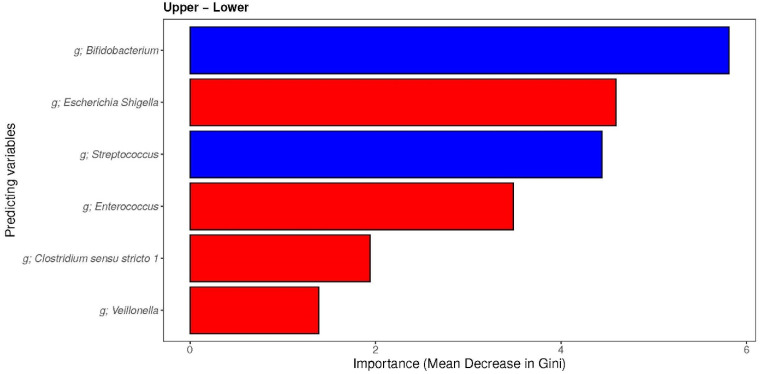
Random forest (RF) analysis indicating which genera are the most important in the “upper” and “lower” MDI-med groups (adjusted FDR analysis). Figure legend: FDR: false discovery rate; red variables were highly associated with the “upper” group (healthier), whereas blue variables were highly associated with the “lower” group (less healthy).

Finally, the prediction of factors of the microbiota (Figure 8) that contribute to the different groups of the MDI-med was performed, using random forest classifier analysis. The models showed good performance on distinguishing the “upper” group from the “lower” group by combining only diet parameters assessed by the MDI-med. The Receiver Operating Characteristic (ROC) curves of the models showed an Area Under the Curve (AUCs) of 0.66 (95% CI: 0.50–0.82) for the MDI-med groups in “upper” vs. “lower”. Important predictors associated with “upper” diet scores were those related to *Bifidobacterium* and *Streptococcus* genus, all of which were described from greatest to least importance using the mean Gini index. The predictors associated with the “lower” MDI-med group were *E. Shigella, Enterococcus, Clostridium sensu stricto 1*, and *Veillonella* genus, also described from greatest to least importance.

## 4. Discussion

The MDI was developed and validated in a US population (MDI-US) of pregnant women, which showed significant reductions in offspring allergies by 4 years of age. To extrapolate the use of the MDI-US in other populations and to investigate its association with the infant gut microbiota at 1 month, we created the MDI-meds using data from the MAMI cohort. We found that an “upper” MDI-med score was characterized by a higher daily vegetable intake and that it was associated with reduced infant microbial diversity (Shannon and Inv Simpson indices scores), but not with microbial richness (Chao1 index), at 1 month of age. In addition, higher maternal intakes of vegetables and yogurt were associated with higher relative abundances of the *Bifidobacterium* genus in infant guts.

The infant gut microbiota is highly dynamic, with visible changes seen in the composition of the gut bacteria, particularly in the first 2 years of life [19]. This constant change in the gut microbiome can be influenced by numerous factors, such as maternal [2] and infant nutrition [19]. In agreement with our results, the infant gut in the first few months of life is characterized by lower microbial diversity [2,16], mainly due to the higher prevalence of *Bifidobacterium* species [19]. It was, therefore, reassuring that the MDI-med was associated with reduced infant microbial diversity at one month, using the alpha diversity measures of Shannon and InvSimpson indices in the adjusted models. This also supports the findings of Fan et al. [6], showing a significant association between maternal vegetable consumption and alpha diversity measured with the Shannon, InvSimpson, and Chao1 indices. Interestingly, we found distinct infant microbiota profiles for the “upper” and “lower” MDI-med groups, but no significant differences (*p* = 0.076). However, combining lactation and the MDI clusters in the joint PERMANOVA analysis showed a significant association, indicating that, together, MDI-med and lactation have a significant impact on the infant gut microbial community’s composition. When we compared Euclidian distance and Bray–Curtis distance, we noticed a significant association between vegetable intake in the “lower” group and beta diversity in the adjusted models, indicating that, together, they account for 3.2% of the composition variability.

Very little data are available on individual food or food group consumption during pregnancy and infant gut microbiomes. In terms of species of importance in the early infant gut microbiome, *Bifidobacterium* members dominate in the microbiome of breastfed and vaginally born infants. Merter et al. [15] and others [14] also described an abundance of *Lactobacillus* and *Streptococcus* in breastfed infants. Low microbial diversity and the predominance of *Bifidobacterium* members are considered characteristic traits of early microbiota composition [15]. The *Bifidobacterium* species in infants is considered to promote the maturation of the healthy immune system.

Previous data from the MAMI cohort (*n* = 116) cohort [2], using information on nutrient intake rather than food consumption, showed several significant associations. The maternal microbiota at delivery time was associated with diet patterns. Two distinct microbial clusters were identified: the first was characterized by *Prevotella* (Cluster I) and higher intakes of total dietary fiber, omega-3 fatty acids, and polyphenols, and the second was characterized by the *Ruminococcus* genus (Cluster II). Neonatal bacterial diversity, measured by the Shannon index, was negatively correlated with maternal vegetable, protein, and fiber intakes. C-section infants, born from mothers with lower fiber intake and *n*-3 fatty acids and higher intakes of animal protein and saturated fatty acids (SFAs), had a microbiota enriched with *Prevotella*, followed by the *Peptoniphilus, Anaerococcus,* and *Porphyromonas* genera [3]. In addition, these genera also showed a negative correlation with fiber and vegetable intake. Total carbohydrate, vegetable, and fiber intakes were negatively associated with Firmicutes phylum members. However, in the C-section-born infants, the authors reported that the offspring of mothers classified as higher fiber and poly-unsaturated fatty acid consumers showed a higher relative abundance of the Proteobacteria phylum (now referred to as Pseudomonadota). Maternal poly-unsaturated fatty acids (PUFA) consumption was negatively associated with the Firmicutes phylum (now referred to as the Bacillota phylum). At the genus level, the MAMI team reported correlations between neonatal microbiota and maternal protein intake. Protein intake from animal sources particularly correlated with *Veillonella*, *Escherichia/Shigella*, *Klebsiella*, or *Clostridium* genus.

In another subset from the MAMI cohort (*n* = 73) [2], the researchers reported that Firmicutes in the neonatal gut microbiota were positively associated with maternal fat intake, especially SFAs and monounsaturated fatty acids (MUFAs), and negatively correlated with the intake of fiber, vegetables, proteins, and vitamins. The team also reported that fat intake (SFAs and MUFAs) was related to higher intestinal permeability, measured by zonulin and intestinal alkaline phosphatase (IAP), and to an adult-type microbiota in the neonatal fecal samples.

Our RDA data show that vegetable and yogurt intake (shown as positive scores in our MDI-med) was associated with the *Bifidobacterium* species. “Upper” MDI-med scores were also associated with higher levels of *Bifidobacterium* and *Streptococcus* members. Similarly, the Spearman correlations indicated significant positive correlations particularly for daily vegetable intake in the “upper” group with the genus *Bifidobacterium* (*p* = 0.01). In contrast, in the “lower” group, daily vegetable intake showed significant negative correlations with the genera *Anaerococcus*, *Peptoniphilus*, *Phascolarctobacterium*, and *Subdoligranulum* (*p* = 0.01). Daily pure fruit juice intake also showed a negative association with *Peptoniphilus* (*p* = 0.01). These bacteria are often associated with infections and may increase due to a higher fat intake [20]. Their role in the infant gut microbiome has not been established.

The random forest (RF) analysis indicated that foods associated with negative scores in the MDI-med included rice and low-fiber grains (carbohydrate intake), red meat, fried potato, low-fiber cereal, and fruit juice. Moreover, the intake of rice and low-fiber grains was associated with *E. Shigella* (*Enterobacteriaceae* family) and *Enterococcus* (*Enterobacteriaceae* family). *Streptococcus* (*Streptococcaceae* family), *Lactobacillus* (*Lactobacillaceae* family), *Ruminococcus gnavus* (Clostridia class), and *Bacteroides* (Bacteroidia class) were associated with the daily intake of red meat, fried potato products, and cold and low-fiber cereal. *Erysipelatoclostridium* (Clostridia class) and *Staphylococcus* (*Staphylococcaceae* family) showed an association with daily pure fruit juice intake. “Lower” MDI-med scores were also associated with increased numbers of *E.coli*, *Shigella*, *Enterococcus*, *Clostridium sensu stricto 1*, and *Veillonella*. Many of these bacteria are often found in fecal samples of formula-fed infants [14,15]. Lundgren et al. [21] reported an association between increased fruit intake and increased odds of increased *Streptococcus/Clostridium* among infants who were 6 weeks of age and born vaginally. With this methodology, we have seen the same associations regarding fruit juice intake, but also with the intake of red meat, fried potato products, and cold cereal. They also reported higher levels of *Clostridium* in C-section-born infants whose mothers showed an increased intake of dairy [21], which was not seen with yogurt consumption in our study.

## 5. Study Limitations

We noticed limitations in the study. As this was an observational study, we had to create extreme scores of the MDI-med, in order to create scores like those we would expect to see in a randomized controlled trial, limiting our study numbers. Furthermore, it must be taken into consideration that, irrespective of the food group, there may be differences in the foods mainly consumed in the two populations, i.e., US vs. Spain. Factors such as the differences in the production of yoghurts, the type of vegetables, or the fat in which French fries are cooked may have influenced the observations made. Also, portion sizes were not included in either the MDI-US or the MDI-med. In the US study, the MDI was associated with reduced infant allergy outcomes up to four years of age [1], but the number of allergy outcomes in the MDI-med were insufficient to study any associations. The strength of the study is the novel data provided here, giving some preliminary associations between maternal overall diet, food group, food intake, and the infant microbiome at one month of age.

## 6. Conclusions

MDI-med scores were associated with maternal diet intake in pregnancy, as reported for MDI-US. Maternal diet during pregnancy influences the infant microbiota. We observed, for the “upper” MDI-med scores, reduced infant gut microbial diversity at one month and, also, specific associations with microbial traits, such as *Bifidobacterium* members. The maternal intake of vegetables and yogurt was a characteristic of the “upper” MDI-med score and, also, it was associated with the presence of the *Bifidobacterium* genus. Our study provides preliminary data on the associations between maternal diet during pregnancy and early infant gut microbiota; further studies with a larger sample size and allergy-related and other health outcomes are needed.

## Figures and Tables

**Table 1:** Characteristics of study participants by MDI-med dichotomy.

	Total (N = 45)	Lower (N = 22)	Upper (N = 23)	*p*-Value *
**Maternal characteristics**				
**Maternal age** *mean (sd)*	34.71 (3.99)	35.22 (3.98)	34.18 (4.03)	0.39
**Weight gain during pregnancy (kg)** *mean (sd)*	12.54 (3.70)	13.39 (3.40)	11.66 (3.87)	0.12
**Pre-pregnancy BMI** *median (sd)*	23.73 (3.43)	23.55 (3.29)	23.92 (3.64)	0.72
**Education**				1
Basic education (mandatory school)	4 (8.9%)	2 (8.7%)	2 (9.1%)	
Medium	11 (24.4%)	6 (26.1%)	5 (22.7%)	
Higher education (university studies)	30 (66.7%)	15 (65.2%)	15 (68.2%)	
**Gestational age (weeks)** *median (IQR)*	40 (39–40)	40 (38–40)	40 (39–40)	0.19
**Antibiotic during pregnancy** *prevalence (%)*	10 (22.2%)	7 (30.4%)	3 (13.6%)	0.67
**Antibiotic intrapartum** *prevalence (%)*	18 (40.0%)	12 (52.2%)	6 (27.3%)	0.16
**Mode of delivery** *prevalence (%)*				0.27
Vaginal	28 (62.2%)	12 (52.2%)	16 (72.7%)	
C-section	17 (37.8%)	11 (47.8%)	6 (27.3%)	
**Neonatal Characteristics**				
**Neonate sex** *prevalence (%)*				0.66
Female	23 (51.1%)	13 (56.5%)	10 (45.5%)	
Male	22 (48.9%)	10 (43.5%)	12 (54.5%)	
**Neonatal height at birth (cm)** *mean (sd)*	50.07 (2.25)	49.09 (2.18)	51.09 (1.86)	0.002
**Neonatal weight at birth (kg)** *mean (sd)*	3.36 (0.48)	3.24 (0.51)	3.48 (0.43)	0.10
**Breastfeeding practices at 1 month** *prevalence (%)*				0.17
Exclusively breast milk	29 (64.4%)	13 (56.5%)	16 (72.7%)	
Infant formula	4 (8.9%)	4 (17.4%)	0 (0.0%)	
Mixed (breast milk and formula)	12 (26.7%)	6 (26.1%)	6 (27.3%)	

Clinical characteristics are represented in bold and values representing the data values are in italics. * Continuous variable differences were assessed using *T*-test and Mann–Whitney analyses, according to data normality assessed by the Shapiro–Wilk test. A chi-squared test was used to assess the significance of the differences in the population’s categorical variables. *p* < 0.05 was considered statistically significant.

**Table 2:** Linear regression estimates of the MDI-med “upper” group compared to the “lower” group using alpha diversity measures for 1-month-old infant microbiomes.

	Unadjusted Model		Adjusted Model 1 ^+^		Adjusted Model 2 ^++^	
Outcome	Estimate (95% CI)	*p*-Value	Estimate (95% CI)	*p*-Value	Estimate (95% CI)	*p*-Value
Shannon Index	−0.35 (−0.66, −0.04)	0.03 *	−0.33 (−0.66, −0.01)	0.04 *	−0.33 (−0.68, 0.03)	0.07
Inverse Simpson	−1.99 (−3.8, −0.18)	0.03 *	−1.78 (−3.62, 0.07)	0.06	−1.42 (−3.35, 0.52)	0.15
Chao1 Index	−3.9 (−18.02, 10.23)	0.58	−3.32 (−17.93, 11.29)	0.65	1.56 (−12.91, 16.04)	0.83

Table legend: ^+^ adjusted model 1 includes only the mode of birth and intrapartum antibiotic; ^++^ adjusted model 2 includes mode of birth, lactation type at 1 month, and maternal gestational antibiotics use. * *p*-value < 0.05 indicates significant differences between groups.

## Data Availability

Data are contained within the article.

## Appendix A

**Table A1:** Comparison of original HS questions and the MAMI FFQ.

Index Components	Healthy Start	MAMI FFQ Responses Included	English Translation of MAMI FFQ Responses
Vegetables	Vegetables: How often did you eat **other vegetables**, NOT INCLUDING what you just told me about (lettuce salads, potatoes, or cooked dried beans)	Espinaca Brécol Lechuga Tomate Zanahorias Judía verde Calabacín Pimiento rojo Espárragos Alcachofa Cebolla Ajo Perejil Champiñón	Spinach Broccoli Lettuce Tomato Carrots Green bean Courgette Red pepper Asparagus Artichoke Onion Garlic Parsley Mushroom
Yogurt	Yogurt: How often did you eat **yogurt** (NOT including frozen yogurt)?	Yogurt entero Yogurt desnatado	Whole yogurt Skimmed yogurt
Fried potato	Fried potatoes: How often did you eat **French fries**, **home fries**, **hash browned potatoes**, or **tater tots**?	Patata frita casera	Fried potato
Fruit juice	Pure juice: How often did you drink **100% pure fruit juice** such as orange, mango, apple, grape and pineapple juices? Do not include fruit-flavored drinks with added sugar or fruit juice you made at home and added sugar to.	Zumo envase	Commercial juice
Cereals	Cereal: How often did you eat **cold cereal** (box cereal such as Corn Flakes)?	Cereales desayuno	Breakfast cereals
Rice	Rice/grains: How often did you eat **rice** or **other cooked grains** (such as bulgur, cracked wheat, or millet)?	Arroz blanco	White rice
Meat	Red meat: How often did you eat **red meat**, such as beef, pork, ham, or sausage? Do not include chicken, turkey or seafood.	Ternera Cerdo Cordero Jamón serrano Jamón york Embutidos Carne picada Panceta	Beef Pig Lamb Serrano ham York ham Sausages Mince Bacon

## Appendix B

**Table A2:** Daily intake examples of below-median and theoretical diets and their resulting MDI values.

Index Component	Diet 1: Average Intake for Participants with MDI < Median	Diet 2: Theoretical “Perfect” Diet	Diet 3: Theoretical “Better” Diet	Diet 4: Theoretical “ok” Diet
*Components associated with* *allergy prevention*	*← Intake (times/day) →*			
Vegetables	4.9	3	3	1
Yogurt	0.38	1	4/7	4/7
*Components associated with* *increased allergy*	*← Intake (times/day) →*			
Red meat	1	1/7	3/7	3/7
Cold cereals	1.9	0	2/7	2/7
Rice and grains	1	0	2/7	2/7
Fried potatoes	1	1/14	1/7	1/7
100% pure fruit juice	0.29	2/7	4/7	4/7
**Associated MDI value**	**71.94**	**76.35**	**74.80**	**73.05**

## Appendix C

**Table A3:** Demographic summary of all individuals with 1 month microbiome information, based on final inclusion.

	Included (N = 45)	Excluded (N = 75)	Total (N = 120)	*p* Value
**Maternal Age**				
Mean (sd)	34.7 (3.99)	35.0 (4.4)	34.9 (4.2)	0.718
**Weight Gain During Pregnancy (kg)**				
Mean (sd)	12.5 (3.7)	12.4 (4.1)	12.5 (3.95)	0.873
**Pre-pregnancy BMI**				
Median (Q1,Q3)	23.7 (3.4)	22.3 (3.3)	22.9 (3.4)	0.028
**Education**				0.695
Basic school	4 (8.9%)	4 (5.3%)	8 (6.7%)	
medium	11 (24.4%)	17 (22.7%)	28 (23.3%)	
High university	30 (66.7%)	54 (72.0%)	84 (70.0%)	
**Antibiotic during pregnancy**	10 (22.2%)	21 (28.0%)	31 (25.8%)	0.53
**Antibiotic Intrapartum**	18 (40.0%)	18 (24.0%)	36 (30.0%)	0.10
**delivery_type**				0.117
Vaginal delivery	28 (62.2%)	58 (77.3%)	86 (71.7%)	
C-section	17 (37.8%)	17 (22.7%)	34 (28.3%)	
**Allergy/Atopy by 12 months**				0.474
No	32 (71.1%)	59 (78.7%)	91 (75.8%)	
Yes	13 (28.9%)	16 (21.3%)	29 (24.2%)	
**Sex**				0.636
Female	23 (51.1%)	43 (57.3%)	66 (55.0%)	
Male	22 (48.9%)	32 (42.7%)	54 (45.0%)	
**Breast feeding Practices at 1 month**				0.591
breastmilk	29 (64.4%)	52 (69.3%)	81 (67.5%)	
formula	4 (8.9%)	9 (12.0%)	13 (10.8%)	
mixed	12 (26.7%)	14 (18.7%)	26 (21.7%)	
**Neonatal height at birth (cm)**				0.47
Mean (sd)	50.1 (2.2)	49.9 (2.2)	49.99 (2.2)	
**Neonatal weight at birth (kg)**				0.78
Mean (sd)	3.4 (0.5)	3.3 (0.4)	3.3 (0.4)	
**Gestational age (weeks)**				0.48
Median (Q1,Q3)	40 (39, 40)	40 (39, 40)	40 (39, 40)	
